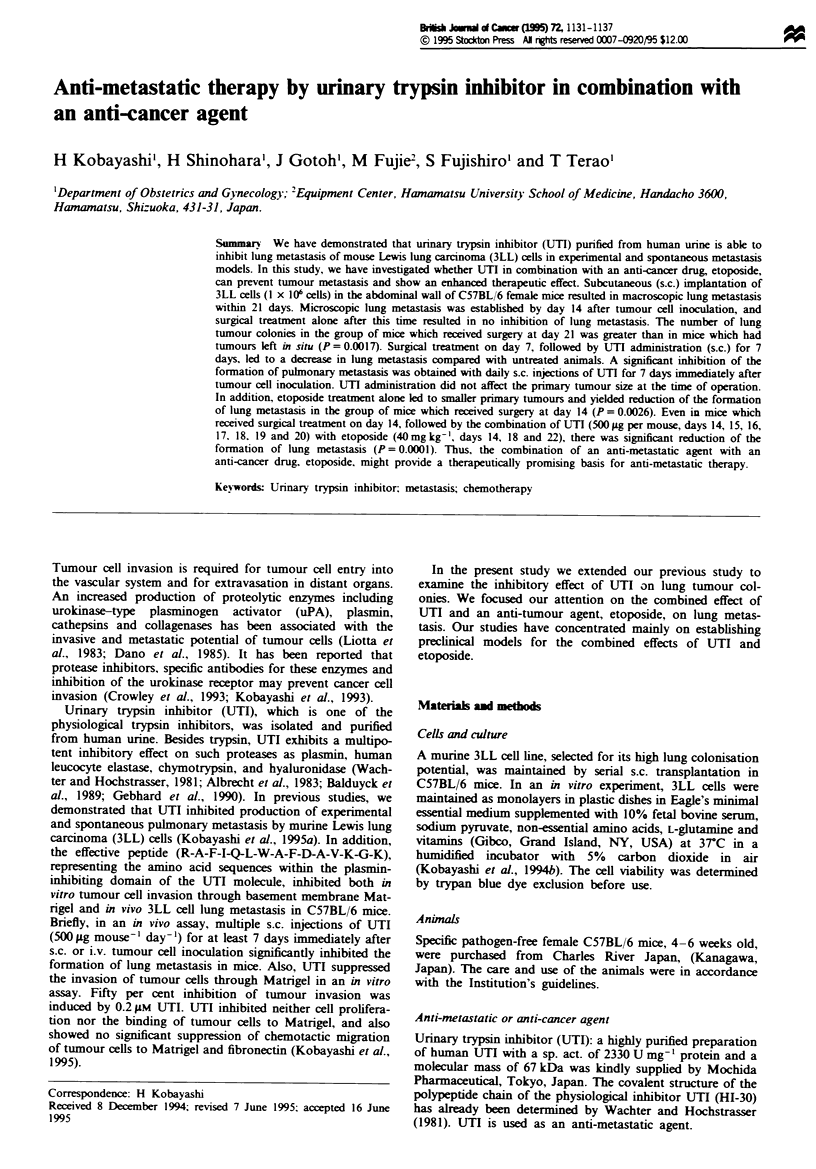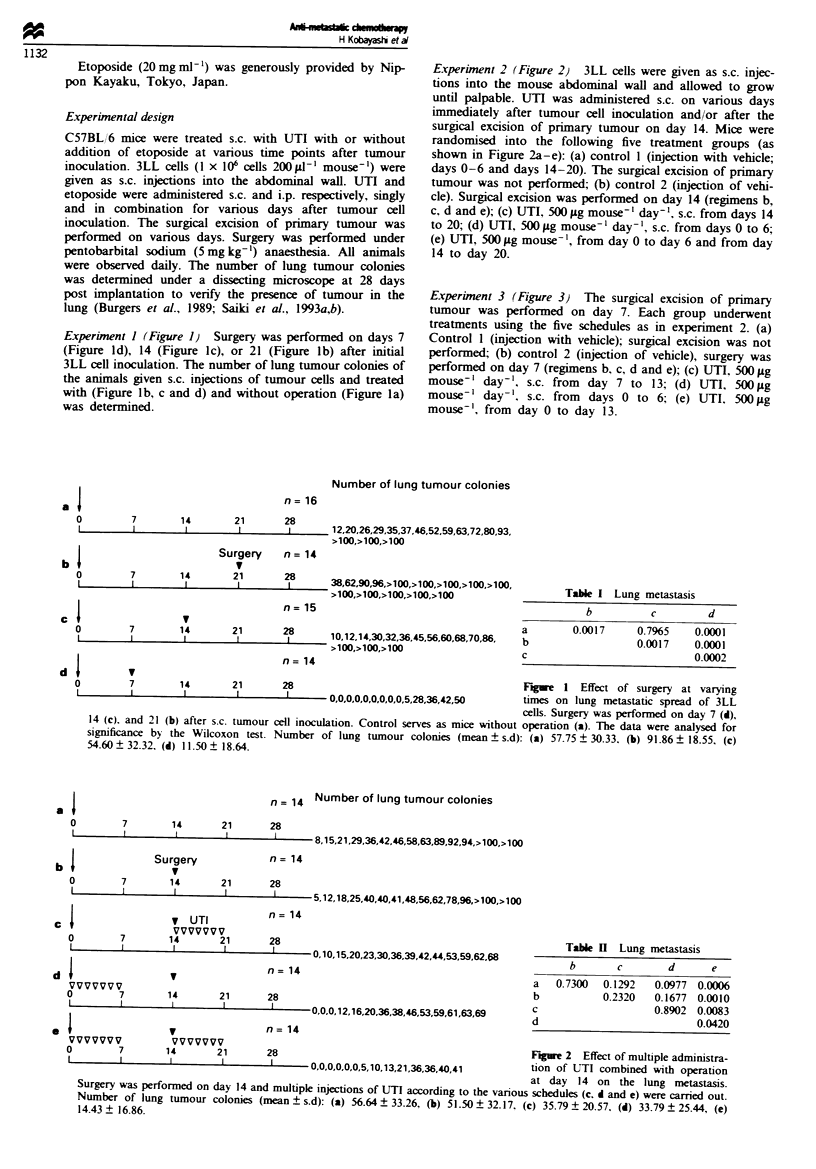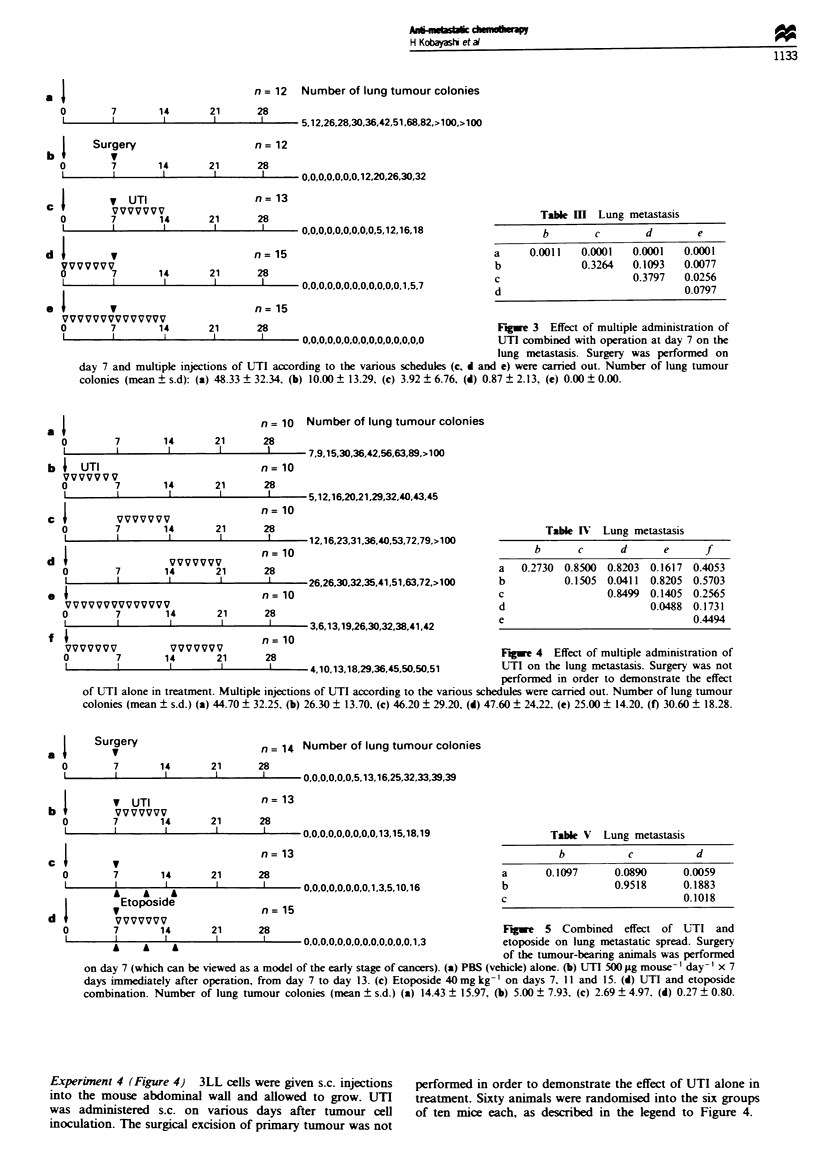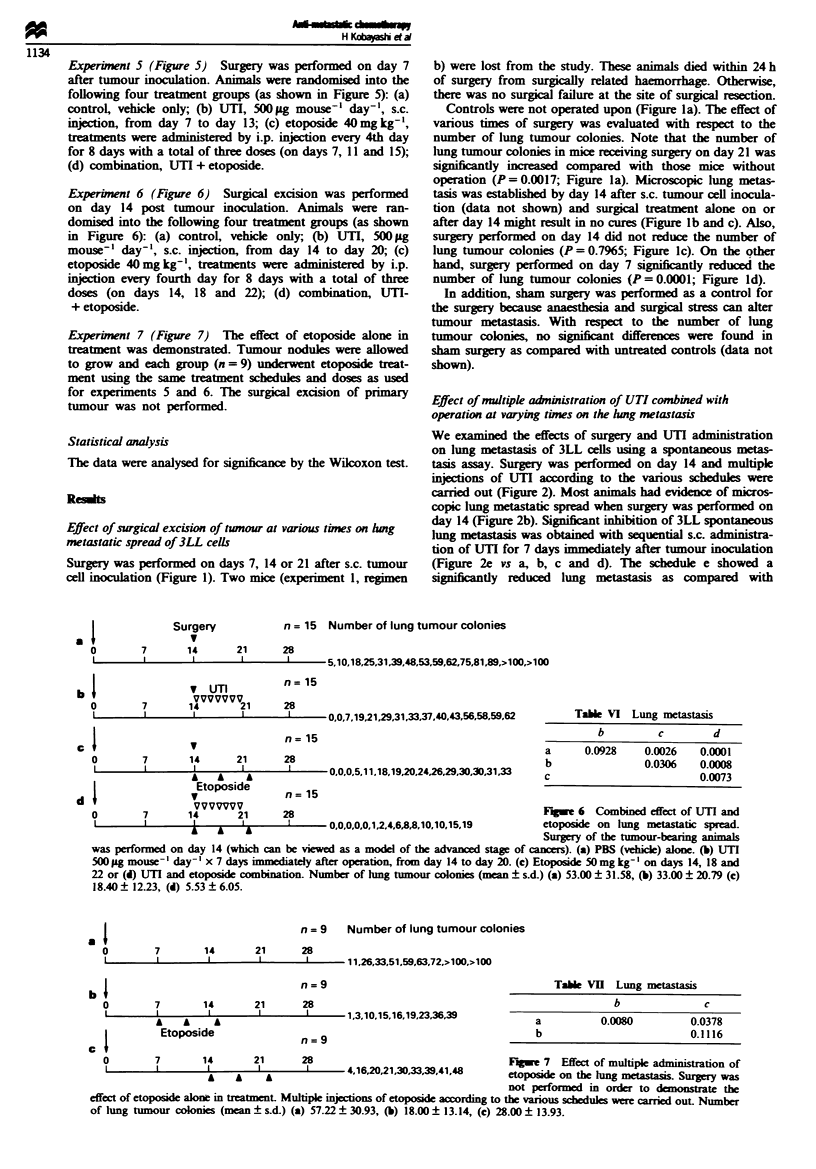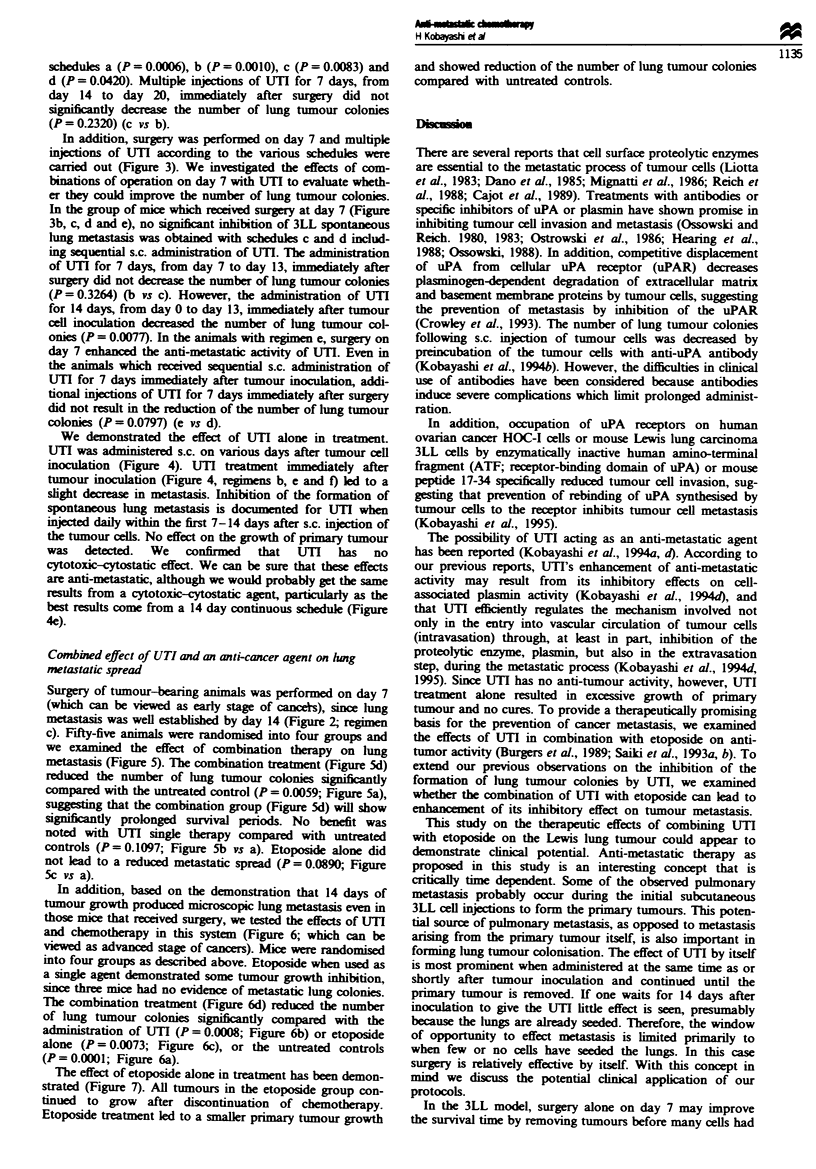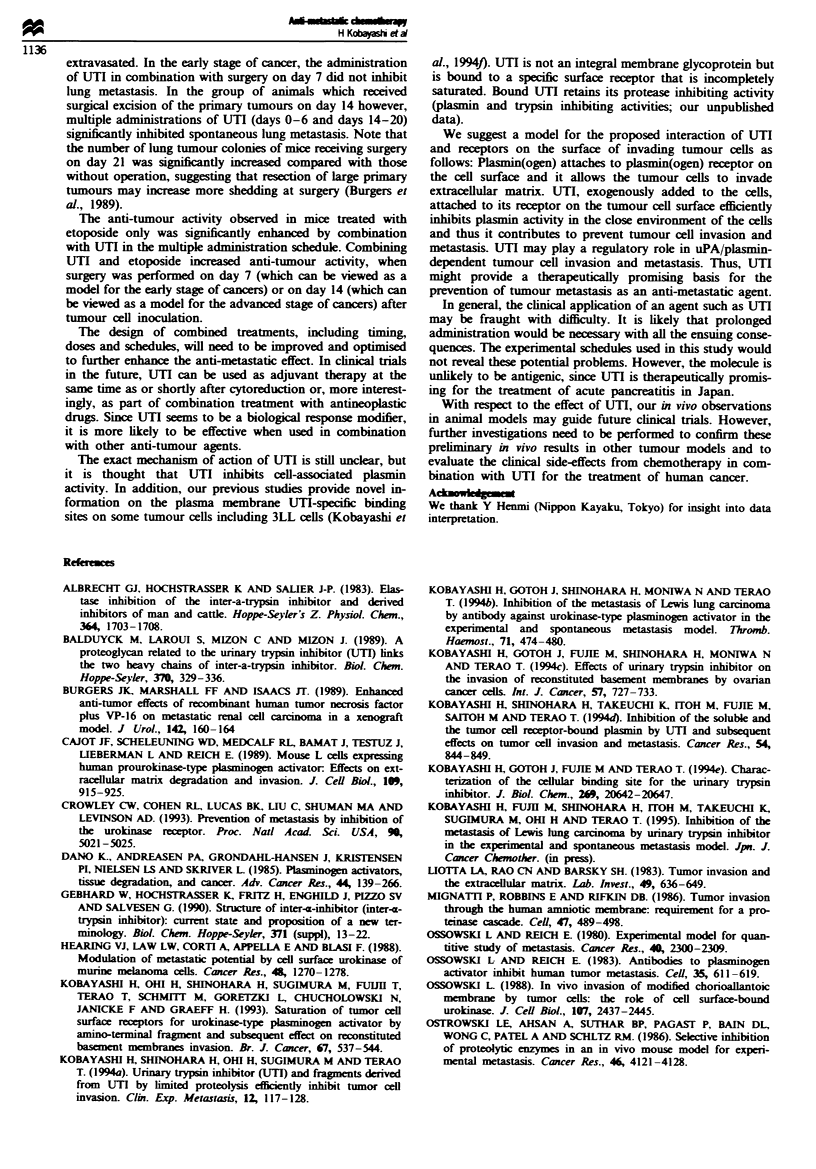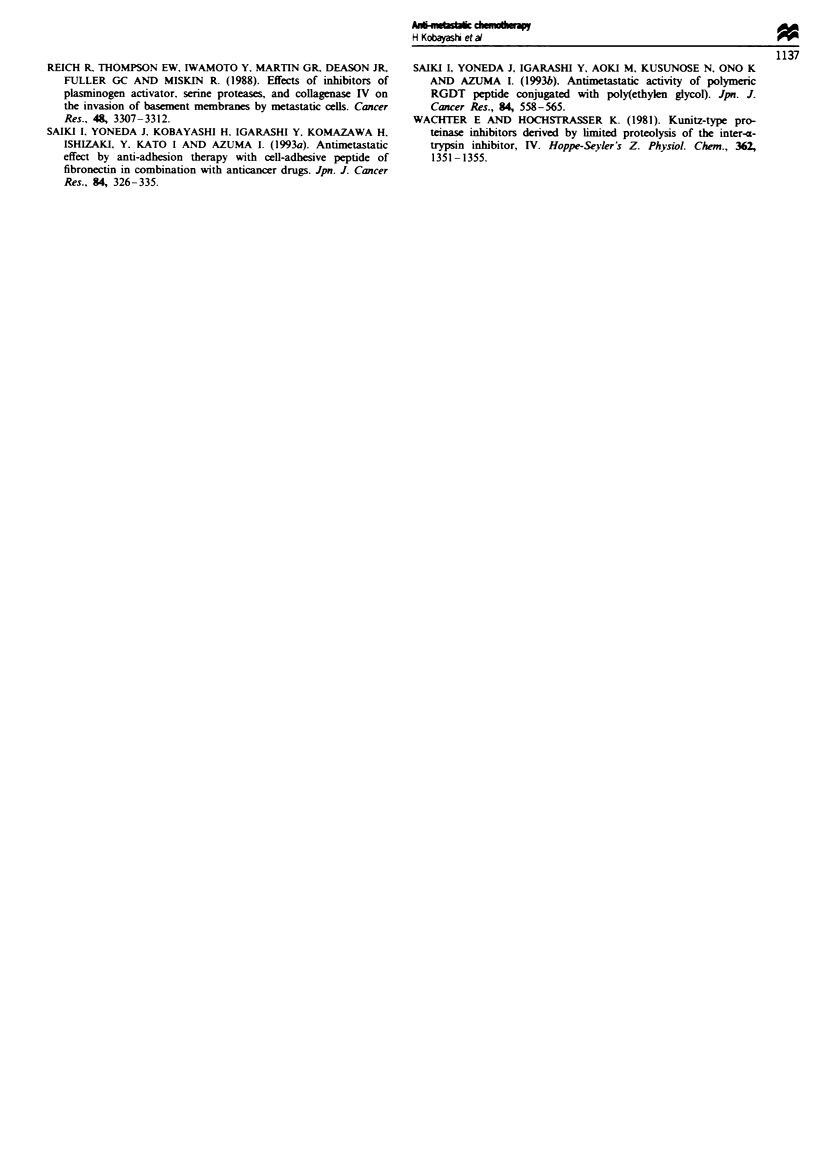# Anti-metastatic therapy by urinary trypsin inhibitor in combination with an anti-cancer agent.

**DOI:** 10.1038/bjc.1995.476

**Published:** 1995-11

**Authors:** H. Kobayashi, H. Shinohara, J. Gotoh, M. Fujie, S. Fujishiro, T. Terao

**Affiliations:** Department of Obstetrics and Gynecology, Hamamatsu University School of Medicine, Shizuoka, Japan.

## Abstract

We have demonstrated that urinary trypsin inhibitor (UTI) purified from human urine is able to inhibit lung metastasis of mouse Lewis lung carcinoma (3LL) cells in experimental and spontaneous metastasis models. In this study, we have investigated whether UTI in combination with an anti-cancer drug, etoposide, can prevent tumour metastasis and show an enhanced therapeutic effect. Subcutaneous (s.c.) implantation of 3LL cells (1 x 10(6) cells) in the abdominal wall of C57BL/6 female mice resulted in macroscopic lung metastasis within 21 days. Microscopic lung metastasis was established by day 14 after tumour cell inoculation, and surgical treatment alone after this time resulted in no inhibition of lung metastasis. The number of lung tumour colonies in the group of mice which received surgery at day 21 was greater than in mice which had tumours left in situ (P = 0.0017). Surgical treatment on day 7, followed by UTI administration (s.c.) for 7 days, led to a decrease in lung metastasis compared with untreated animals. A significant inhibition of the formation of pulmonary metastasis was obtained with daily s.c. injections of UTI for 7 days immediately after tumour cell inoculation. UTI administration did not affect the primary tumour size at the time of operation. In addition, etoposide treatment alone led to a smaller primary tumours and yielded reduction of the formation of lung metastasis in the group of mice which received surgery at day 14 (P = 0.0026). Even in mice which received surgical treatment on day 14, followed by the combination of UTI (500 micrograms per mouse, days 14, 15, 16, 17, 18, 19 and 20) with etoposide (40 mg kg-1, days 14, 18 and 22), there was significant reduction of the formation of lung metastasis (P = 0.0001). Thus, the combination of an anti-metastatic agent with an anti-cancer drug, etoposide, might provide a therapeutically promising basis for anti-metastatic therapy.


© 1995 Stockton Press All rights reserved 0007-0920/95 $12.00

### Anti-metastatic therapy by urinary trypsin inhibitor in combination with an anti-cancer agent

H Kobayashi[1], H Shinohara[1], J Gotoh[1], M Fujie[2], S Fujishiro[1] and T Terao[1]

[1]*Department of Obstetrics and Gynecology;* [2]*Equipment Center, Hamamatsu University School of Medicine, Handacho 3600, Hamamatsu, Shizuoka, 431-31, Japan.*

**Summary** We have demonstrated that urinary trypsin inhibitor (UTI) purified from human urine is able to inhibit lung metastasis of mouse Lewis lung carcinoma (3LL) cells in experimental and spontaneous metastasis models. In this study, we have investigated whether UTI in combination with an anti-cancer drug, etoposide, can prevent tumour metastasis and show an enhanced therapeutic effect. Subcutaneous (s.c.) implantation of 3LL cells ($1 \times 10^6$ cells) in the abdominal wall of C57BL/6 female mice resulted in macroscopic lung metastasis within 21 days. Microscopic lung metastasis was established by day 14 after tumour cell inoculation, and surgical treatment alone after this time resulted in no inhibition of lung metastasis. The number of lung tumour colonies in the group of mice which received surgery at day 21 was greater than in mice which had tumours left *in situ* ($P = 0.0017$). Surgical treatment on day 7, followed by UTI administration (s.c.) for 7 days, led to a decrease in lung metastasis compared with untreated animals. A significant inhibition of the formation of pulmonary metastasis was obtained with daily s.c. injections of UTI for 7 days immediately after tumour cell inoculation. UTI administration did not affect the primary tumour size at the time of operation. In addition, etoposide treatment alone led to smaller primary tumours and yielded reduction of the formation of lung metastasis in the group of mice which received surgery at day 14 ($P = 0.0026$). Even in mice which received surgical treatment on day 14, followed by the combination of UTI (500 μg per mouse, days 14, 15, 16, 17, 18, 19 and 20) with etoposide (40 mg $kg^{-1}$, days 14, 18 and 22), there was significant reduction of the formation of lung metastasis ($P = 0.0001$). Thus, the combination of an anti-metastatic agent with an anti-cancer drug, etoposide, might provide a therapeutically promising basis for anti-metastatic therapy.

**Keywords:** Urinary trypsin inhibitor; metastasis; chemotherapy

Tumour cell invasion is required for tumour cell entry into the vascular system and for extravasation in distant organs. An increased production of proteolytic enzymes including urokinase-type plasminogen activator (uPA), plasmin, cathepsins and collagenases has been associated with the invasive and metastatic potential of tumour cells (Liotta *et al.*, 1983; Dano *et al.*, 1985). It has been reported that protease inhibitors, specific antibodies for these enzymes and inhibition of the urokinase receptor may prevent cancer cell invasion (Crowley *et al.*, 1993; Kobayashi *et al.*, 1993).

Urinary trypsin inhibitor (UTI), which is one of the physiological trypsin inhibitors, was isolated and purified from human urine. Besides trypsin, UTI exhibits a multipotent inhibitory effect on such proteases as plasmin, human leucocyte elastase, chymotrypsin, and hyaluronidase (Wachter and Hochstrasser, 1981; Albrecht *et al.*, 1983; Balduyck *et al.*, 1989; Gebhard *et al.*, 1990). In previous studies, we demonstrated that UTI inhibited production of experimental and spontaneous pulmonary metastasis by murine Lewis lung carcinoma (3LL) cells (Kobayashi *et al.*, 1995*a*). In addition, the effective peptide (R-A-F-I-Q-L-W-A-F-D-A-V-K-G-K), representing the amino acid sequences within the plasmin-inhibiting domain of the UTI molecule, inhibited both *in vitro* tumour cell invasion through basement membrane Matrigel and *in vivo* 3LL cell lung metastasis in C57BL/6 mice. Briefly, in an *in vivo* assay, multiple s.c. injections of UTI (500 μg $mouse^{-1}$ $day^{-1}$) for at least 7 days immediately after s.c. or i.v. tumour cell inoculation significantly inhibited the formation of lung metastasis in mice. Also, UTI suppressed the invasion of tumour cells through Matrigel in an *in vitro* assay. Fifty per cent inhibition of tumour invasion was induced by 0.2 μM UTI. UTI inhibited neither cell proliferation nor the binding of tumour cells to Matrigel, and also showed no significant suppression of chemotactic migration of tumour cells to Matrigel and fibronectin (Kobayashi *et al.*, 1995).

Correspondence: H Kobayashi
Received 8 December 1994; revised 7 June 1995; accepted 16 June 1995

In the present study we extended our previous study to examine the inhibitory effect of UTI on lung tumour colonies. We focused our attention on the combined effect of UTI and an anti-tumour agent, etoposide, on lung metastasis. Our studies have concentrated mainly on establishing preclinical models for the combined effects of UTI and etoposide.

### Materials and methods

### *Cells and culture*

A murine 3LL cell line, selected for its high lung colonisation potential, was maintained by serial s.c. transplantation in C57BL/6 mice. In an *in vitro* experiment, 3LL cells were maintained as monolayers in plastic dishes in Eagle's minimal essential medium supplemented with 10% fetal bovine serum, sodium pyruvate, non-essential amino acids, L-glutamine and vitamins (Gibco, Grand Island, NY, USA) at 37°C in a humidified incubator with 5% carbon dioxide in air (Kobayashi *et al.*, 1994*b*). The cell viability was determined by trypan blue dye exclusion before use.

### *Animals*

Specific pathogen-free female C57BL/6 mice, 4–6 weeks old, were purchased from Charles River Japan, (Kanagawa, Japan). The care and use of the animals were in accordance with the Institution's guidelines.

### *Anti-metastatic or anti-cancer agent*

Urinary trypsin inhibitor (UTI): a highly purified preparation of human UTI with a sp. act. of 2330 U $mg^{-1}$ protein and a molecular mass of 67 kDa was kindly supplied by Mochida Pharmaceutical, Tokyo, Japan. The covalent structure of the polypeptide chain of the physiological inhibitor UTI (HI-30) has already been determined by Wachter and Hochstrasser (1981). UTI is used as an anti-metastatic agent.

Etoposide (20 mg $ml^{-1}$) was generously provided by Nippon Kayaku, Tokyo, Japan.

*Experimental design*

C57BL/6 mice were treated s.c. with UTI with or without addition of etoposide at various time points after tumour inoculation. 3LL cells ($1 \times 10^6$ cells 200 $\mu l^{-1}$ $mouse^{-1}$) were given as s.c. injections into the abdominal wall. UTI and etoposide were administered s.c. and i.p. respectively, singly and in combination for various days after tumour cell inoculation. The surgical excision of primary tumour was performed on various days. Surgery was performed under pentobarbital sodium (5 mg $kg^{-1}$) anaesthesia. All animals were observed daily. The number of lung tumour colonies was determined under a dissecting microscope at 28 days post implantation to verify the presence of tumour in the lung (Burgers *et al.*, 1989; Saiki *et al.*, 1993*a*,*b*).

*Experiment 1 (Figure 1)* Surgery was performed on days 7 (Figure 1d), 14 (Figure 1c), or 21 (Figure 1b) after initial 3LL cell inoculation. The number of lung tumour colonies of the animals given s.c. injections of tumour cells and treated with (Figure 1b, c and d) and without operation (Figure 1a) was determined.

*Experiment 2 (Figure 2)* 3LL cells were given as s.c. injections into the mouse abdominal wall and allowed to grow until palpable. UTI was administered s.c. on various days immediately after tumour cell inoculation and/or after the surgical excision of primary tumour on day 14. Mice were randomised into the following five treatment groups (as shown in Figure 2a–e): (a) control 1 (injection with vehicle; days 0–6 and days 14–20). The surgical excision of primary tumour was not performed; (b) control 2 (injection of vehicle). Surgical excision was performed on day 14 (regimens b, c, d and e); (c) UTI, 500 $\mu g$ $mouse^{-1}$ $day^{-1}$, s.c. from days 14 to 20; (d) UTI, 500 $\mu g$ $mouse^{-1}$ $day^{-1}$, s.c. from days 0 to 6; (e) UTI, 500 $\mu g$ $mouse^{-1}$, from day 0 to day 6 and from day 14 to day 20.

*Experiment 3 (Figure 3)* The surgical excision of primary tumour was performed on day 7. Each group underwent treatments using the five schedules as in experiment 2. (a) Control 1 (injection with vehicle); surgical excision was not performed; (b) control 2 (injection of vehicle), surgery was performed on day 7 (regimens b, c, d and e); (c) UTI, 500 $\mu g$ $mouse^{-1}$ $day^{-1}$, s.c. from day 7 to 13; (d) UTI, 500 $\mu g$ $mouse^{-1}$ $day^{-1}$, s.c. from days 0 to 6; (e) UTI, 500 $\mu g$ $mouse^{-1}$, from day 0 to day 13.

**Table I** Lung metastasis

| | b | c | d |
|---|---|---|---|
| a | 0.0017 | 0.7965 | 0.0001 |
| b | | 0.0017 | 0.0001 |
| c | | | 0.0002 |

**Figure 1** Effect of surgery at varying times on lung metastatic spread of 3LL cells. Surgery was performed on day 7 (**d**), 14 (**c**), and 21 (**b**) after s.c. tumour cell inoculation. Control serves as mice without operation (**a**). The data were analysed for significance by the Wilcoxon test. Number of lung tumour colonies (mean ± s.d): (**a**) 57.75 ± 30.33, (**b**) 91.86 ± 18.55, (**c**) 54.60 ± 32.32, (**d**) 11.50 ± 18.64.

**Table II** Lung metastasis

| | b | c | d | e |
|---|---|---|---|---|
| a | 0.7300 | 0.1292 | 0.0977 | 0.0006 |
| b | | 0.2320 | 0.1677 | 0.0010 |
| c | | | 0.8902 | 0.0083 |
| d | | | | 0.0420 |

**Figure 2** Effect of multiple administration of UTI combined with operation at day 14 on the lung metastasis. Surgery was performed on day 14 and multiple injections of UTI according to the various schedules (**c**, **d** and **e**) were carried out. Number of lung tumour colonies (mean ± s.d): (**a**) 56.64 ± 33.26, (**b**) 51.50 ± 32.17, (**c**) 35.79 ± 20.57, (**d**) 33.79 ± 25.44, (**e**) 14.43 ± 16.86.

**Table III** Lung metastasis

| | *b* | *c* | *d* | *e* |
|---|---|---|---|---|
| a | 0.0011 | 0.0001 | 0.0001 | 0.0001 |
| b | | 0.3264 | 0.1093 | 0.0077 |
| c | | | 0.3797 | 0.0256 |
| d | | | | 0.0797 |

**Figure 3** Effect of multiple administration of UTI combined with operation at day 7 on the lung metastasis. Surgery was performed on day 7 and multiple injections of UTI according to the various schedules (**c**, **d** and **e**) were carried out. Number of lung tumour colonies (mean ± s.d): (**a**) 48.33 ± 32.34, (**b**) 10.00 ± 13.29, (**c**) 3.92 ± 6.76, (**d**) 0.87 ± 2.13, (**e**) 0.00 ± 0.00.

**Table IV** Lung metastasis

| | *b* | *c* | *d* | *e* | *f* |
|---|---|---|---|---|---|
| a | 0.2730 | 0.8500 | 0.8203 | 0.1617 | 0.4053 |
| b | | 0.1505 | 0.0411 | 0.8205 | 0.5703 |
| c | | | 0.8499 | 0.1405 | 0.2565 |
| d | | | | 0.0488 | 0.1731 |
| e | | | | | 0.4494 |

**Figure 4** Effect of multiple administration of UTI on the lung metastasis. Surgery was not performed in order to demonstrate the effect of UTI alone in treatment. Multiple injections of UTI according to the various schedules were carried out. Number of lung tumour colonies (mean ± s.d.) (**a**) 44.70 ± 32.25, (**b**) 26.30 ± 13.70, (**c**) 46.20 ± 29.20, (**d**) 47.60 ± 24.22, (**e**) 25.00 ± 14.20, (**f**) 30.60 ± 18.28.

**Table V** Lung metastasis

| | *b* | *c* | *d* |
|---|---|---|---|
| a | 0.1097 | 0.0890 | 0.0059 |
| b | | 0.9518 | 0.1883 |
| c | | | 0.1018 |

**Figure 5** Combined effect of UTI and etoposide on lung metastatic spread. Surgery of the tumour-bearing animals was performed on day 7 (which can be viewed as a model of the early stage of cancers). (**a**) PBS (vehicle) alone. (**b**) UTI 500 μg mouse$^{-1}$ day$^{-1}$ × 7 days immediately after operation, from day 7 to day 13. (**c**) Etoposide 40 mg kg$^{-1}$ on days 7, 11 and 15. (**d**) UTI and etoposide combination. Number of lung tumour colonies (mean ± s.d.) (**a**) 14.43 ± 15.97, (**b**) 5.00 ± 7.93, (**c**) 2.69 ± 4.97, (**d**) 0.27 ± 0.80.

*Experiment 4 (Figure 4)* 3LL cells were given s.c. injections into the mouse abdominal wall and allowed to grow. UTI was administered s.c. on various days after tumour cell inoculation. The surgical excision of primary tumour was not performed in order to demonstrate the effect of UTI alone in treatment. Sixty animals were randomised into the six groups of ten mice each, as described in the legend to Figure 4.

*Experiment 5 (Figure 5)* Surgery was performed on day 7 after tumour inoculation. Animals were randomised into the following four treatment groups (as shown in Figure 5): (a) control, vehicle only; (b) UTI, 500 μg $mouse^{-1}$ $day^{-1}$, s.c. injection, from day 7 to day 13; (c) etoposide 40 mg $kg^{-1}$, treatments were administered by i.p. injection every 4th day for 8 days with a total of three doses (on days 7, 11 and 15); (d) combination, UTI + etoposide.

*Experiment 6 (Figure 6)* Surgical excision was performed on day 14 post tumour inoculation. Animals were randomised into the following four treatment groups (as shown in Figure 6): (a) control, vehicle only; (b) UTI, 500 μg $mouse^{-1}$ $day^{-1}$, s.c. injection, from day 14 to day 20; (c) etoposide 40 mg $kg^{-1}$, treatments were administered by i.p. injection every fourth day for 8 days with a total of three doses (on days 14, 18 and 22); (d) combination, UTI-+ etoposide.

*Experiment 7 (Figure 7)* The effect of etoposide alone in treatment was demonstrated. Tumour nodules were allowed to grow and each group ($n = 9$) underwent etoposide treatment using the same treatment schedules and doses as used for experiments 5 and 6. The surgical excision of primary tumour was not performed.

### *Statistical analysis*

The data were analysed for significance by the Wilcoxon test.

### Results

### *Effect of surgical excision of tumour at various times on lung metastatic spread of 3LL cells*

Surgery was performed on days 7, 14 or 21 after s.c. tumour cell inoculation (Figure 1). Two mice (experiment 1, regimen b) were lost from the study. These animals died within 24 h of surgery from surgically related haemorrhage. Otherwise, there was no surgical failure at the site of surgical resection.

Controls were not operated upon (Figure 1a). The effect of various times of surgery was evaluated with respect to the number of lung tumour colonies. Note that the number of lung tumour colonies in mice receiving surgery on day 21 was significantly increased compared with those mice without operation ($P = 0.0017$; Figure 1a). Microscopic lung metastasis was established by day 14 after s.c. tumour cell inoculation (data not shown) and surgical treatment alone on or after day 14 might result in no cures (Figure 1b and c). Also, surgery performed on day 14 did not reduce the number of lung tumour colonies ($P = 0.7965$; Figure 1c). On the other hand, surgery performed on day 7 significantly reduced the number of lung tumour colonies ($P = 0.0001$; Figure 1d).

In addition, sham surgery was performed as a control for the surgery because anaesthesia and surgical stress can alter tumour metastasis. With respect to the number of lung tumour colonies, no significant differences were found in sham surgery as compared with untreated controls (data not shown).

### *Effect of multiple administration of UTI combined with operation at varying times on the lung metastasis*

We examined the effects of surgery and UTI administration on lung metastasis of 3LL cells using a spontaneous metastasis assay. Surgery was performed on day 14 and multiple injections of UTI according to the various schedules were carried out (Figure 2). Most animals had evidence of microscopic lung metastatic spread when surgery was performed on day 14 (Figure 2b). Significant inhibition of 3LL spontaneous lung metastasis was obtained with sequential s.c. administration of UTI for 7 days immediately after tumour inoculation (Figure 2e *vs* a, b, c and d). The schedule e showed a significantly reduced lung metastasis as compared with

**Table VI** Lung metastasis

| | *b* | *c* | *d* |
|---|---|---|---|
| a | 0.0928 | 0.0026 | 0.0001 |
| b | | 0.0306 | 0.0008 |
| c | | | 0.0073 |

**Figure 6** Combined effect of UTI and etoposide on lung metastatic spread. Surgery of the tumour-bearing animals was performed on day 14 (which can be viewed as a model of the advanced stage of cancers). (**a**) PBS (vehicle) alone. (**b**) UTI 500 μg $mouse^{-1}$ $day^{-1}$ × 7 days immediately after operation, from day 14 to day 20. (**c**) Etoposide 50 mg $kg^{-1}$ on days 14, 18 and 22 or (**d**) UTI and etoposide combination. Number of lung tumour colonies (mean ± s.d.) (**a**) 53.00 ± 31.58, (**b**) 33.00 ± 20.79 (**c**) 18.40 ± 12.23, (**d**) 5.53 ± 6.05.

**Table VII** Lung metastasis

| | *b* | *c* |
|---|---|---|
| a | 0.0080 | 0.0378 |
| b | | 0.1116 |

**Figure 7** Effect of multiple administration of etoposide on the lung metastasis. Surgery was not performed in order to demonstrate the effect of etoposide alone in treatment. Multiple injections of etoposide according to the various schedules were carried out. Number of lung tumour colonies (mean ± s.d.) (**a**) 57.22 ± 30.93, (**b**) 18.00 ± 13.14, (**c**) 28.00 ± 13.93.

schedules a ($P = 0.0006$), b ($P = 0.0010$), c ($P = 0.0083$) and d ($P = 0.0420$). Multiple injections of UTI for 7 days, from day 14 to day 20, immediately after surgery did not significantly decrease the number of lung tumour colonies ($P = 0.2320$) (c *vs* b).

In addition, surgery was performed on day 7 and multiple injections of UTI according to the various schedules were carried out (Figure 3). We investigated the effects of combinations of operation on day 7 with UTI to evaluate whether they could improve the number of lung tumour colonies. In the group of mice which received surgery at day 7 (Figure 3b, c, d and e), no significant inhibition of 3LL spontaneous lung metastasis was obtained with schedules c and d including sequential s.c. administration of UTI. The administration of UTI for 7 days, from day 7 to day 13, immediately after surgery did not decrease the number of lung tumour colonies ($P = 0.3264$) (b *vs* c). However, the administration of UTI for 14 days, from day 0 to day 13, immediately after tumour cell inoculation decreased the number of lung tumour colonies ($P = 0.0077$). In the animals with regimen e, surgery on day 7 enhanced the anti-metastatic activity of UTI. Even in the animals which received sequential s.c. administration of UTI for 7 days immediately after tumour inoculation, additional injections of UTI for 7 days immediately after surgery did not result in the reduction of the number of lung tumour colonies ($P = 0.0797$) (e *vs* d).

We demonstrated the effect of UTI alone in treatment. UTI was administered s.c. on various days after tumour cell inoculation (Figure 4). UTI treatment immediately after tumour inoculation (Figure 4, regimens b, e and f) led to a slight decrease in metastasis. Inhibition of the formation of spontaneous lung metastasis is documented for UTI when injected daily within the first 7–14 days after s.c. injection of the tumour cells. No effect on the growth of primary tumour was detected. We confirmed that UTI has no cytotoxic–cytostatic effect. We can be sure that these effects are anti-metastatic, although we would probably get the same results from a cytotoxic–cytostatic agent, particularly as the best results come from a 14 day continuous schedule (Figure 4e).

### *Combined effect of UTI and an anti-cancer agent on lung metastatic spread*

Surgery of tumour-bearing animals was performed on day 7 (which can be viewed as early stage of cancers), since lung metastasis was well established by day 14 (Figure 2; regimen c). Fifty-five animals were randomised into four groups and we examined the effect of combination therapy on lung metastasis (Figure 5). The combination treatment (Figure 5d) reduced the number of lung tumour colonies significantly compared with the untreated control ($P = 0.0059$; Figure 5a), suggesting that the combination group (Figure 5d) will show significantly prolonged survival periods. No benefit was noted with UTI single therapy compared with untreated controls ($P = 0.1097$; Figure 5b *vs* a). Etoposide alone did not lead to a reduced metastatic spread ($P = 0.0890$; Figure 5c *vs* a).

In addition, based on the demonstration that 14 days of tumour growth produced microscopic lung metastasis even in those mice that received surgery, we tested the effects of UTI and chemotherapy in this system (Figure 6; which can be viewed as advanced stage of cancers). Mice were randomised into four groups as described above. Etoposide when used as a single agent demonstrated some tumour growth inhibition, since three mice had no evidence of metastatic lung colonies. The combination treatment (Figure 6d) reduced the number of lung tumour colonies significantly compared with the administration of UTI ($P = 0.0008$; Figure 6b) or etoposide alone ($P = 0.0073$; Figure 6c), or the untreated controls ($P = 0.0001$; Figure 6a).

The effect of etoposide alone in treatment has been demonstrated (Figure 7). All tumours in the etoposide group continued to grow after discontinuation of chemotherapy. Etoposide treatment led to a smaller primary tumour growth and showed reduction of the number of lung tumour colonies compared with untreated controls.

### Discussion

There are several reports that cell surface proteolytic enzymes are essential to the metastatic process of tumour cells (Liotta *et al.*, 1983; Dano *et al.*, 1985; Mignatti *et al.*, 1986; Reich *et al.*, 1988; Cajot *et al.*, 1989). Treatments with antibodies or specific inhibitors of uPA or plasmin have shown promise in inhibiting tumour cell invasion and metastasis (Ossowski and Reich. 1980, 1983; Ostrowski *et al.*, 1986; Hearing *et al.*, 1988; Ossowski, 1988). In addition, competitive displacement of uPA from cellular uPA receptor (uPAR) decreases plasminogen-dependent degradation of extracellular matrix and basement membrane proteins by tumour cells, suggesting the prevention of metastasis by inhibition of the uPAR (Crowley *et al.*, 1993). The number of lung tumour colonies following s.c. injection of tumour cells was decreased by preincubation of the tumour cells with anti-uPA antibody (Kobayashi *et al.*, 1994*b*). However, the difficulties in clinical use of antibodies have been considered because antibodies induce severe complications which limit prolonged administration.

In addition, occupation of uPA receptors on human ovarian cancer HOC-I cells or mouse Lewis lung carcinoma 3LL cells by enzymatically inactive human amino-terminal fragment (ATF; receptor-binding domain of uPA) or mouse peptide 17-34 specifically reduced tumour cell invasion, suggesting that prevention of rebinding of uPA synthesised by tumour cells to the receptor inhibits tumour cell metastasis (Kobayashi *et al.*, 1995).

The possibility of UTI acting as an anti-metastatic agent has been reported (Kobayashi *et al.*, 1994*a*, *d*). According to our previous reports, UTI's enhancement of anti-metastatic activity may result from its inhibitory effects on cell-associated plasmin activity (Kobayashi *et al.*, 1994*d*), and that UTI efficiently regulates the mechanism involved not only in the entry into vascular circulation of tumour cells (intravasation) through, at least in part, inhibition of the proteolytic enzyme, plasmin, but also in the extravasation step, during the metastatic process (Kobayashi *et al.*, 1994*d*, 1995). Since UTI has no anti-tumour activity, however, UTI treatment alone resulted in excessive growth of primary tumour and no cures. To provide a therapeutically promising basis for the prevention of cancer metastasis, we examined the effects of UTI in combination with etoposide on anti-tumor activity (Burgers *et al.*, 1989; Saiki *et al.*, 1993*a*, *b*). To extend our previous observations on the inhibition of the formation of lung tumour colonies by UTI, we examined whether the combination of UTI with etoposide can lead to enhancement of its inhibitory effect on tumour metastasis.

This study on the therapeutic effects of combining UTI with etoposide on the Lewis lung tumour could appear to demonstrate clinical potential. Anti-metastatic therapy as proposed in this study is an interesting concept that is critically time dependent. Some of the observed pulmonary metastasis probably occur during the initial subcutaneous 3LL cell injections to form the primary tumours. This potential source of pulmonary metastasis, as opposed to metastasis arising from the primary tumour itself, is also important in forming lung tumour colonisation. The effect of UTI by itself is most prominent when administered at the same time as or shortly after tumour inoculation and continued until the primary tumour is removed. If one waits for 14 days after inoculation to give the UTI little effect is seen, presumably because the lungs are already seeded. Therefore, the window of opportunity to effect metastasis is limited primarily to when few or no cells have seeded the lungs. In this case surgery is relatively effective by itself. With this concept in mind we discuss the potential clinical application of our protocols.

In the 3LL model, surgery alone on day 7 may improve the survival time by removing tumours before many cells had extravasated. In the early stage of cancer, the administration of UTI in combination with surgery on day 7 did not inhibit lung metastasis. In the group of animals which received surgical excision of the primary tumours on day 14 however, multiple administrations of UTI (days 0–6 and days 14–20) significantly inhibited spontaneous lung metastasis. Note that the number of lung tumour colonies of mice receiving surgery on day 21 was significantly increased compared with those without operation, suggesting that resection of large primary tumours may increase more shedding at surgery (Burgers *et al.*, 1989).

The anti-tumour activity observed in mice treated with etoposide only was significantly enhanced by combination with UTI in the multiple administration schedule. Combining UTI and etoposide increased anti-tumour activity, when surgery was performed on day 7 (which can be viewed as a model for the early stage of cancers) or on day 14 (which can be viewed as a model for the advanced stage of cancers) after tumour cell inoculation.

The design of combined treatments, including timing, doses and schedules, will need to be improved and optimised to further enhance the anti-metastatic effect. In clinical trials in the future, UTI can be used as adjuvant therapy at the same time as or shortly after cytoreduction or, more interestingly, as part of combination treatment with antineoplastic drugs. Since UTI seems to be a biological response modifier, it is more likely to be effective when used in combination with other anti-tumour agents.

The exact mechanism of action of UTI is still unclear, but it is thought that UTI inhibits cell-associated plasmin activity. In addition, our previous studies provide novel information on the plasma membrane UTI-specific binding sites on some tumour cells including 3LL cells (Kobayashi *et al.*, 1994*f*). UTI is not an integral membrane glycoprotein but is bound to a specific surface receptor that is incompletely saturated. Bound UTI retains its protease inhibiting activity (plasmin and trypsin inhibiting activities; our unpublished data).

We suggest a model for the proposed interaction of UTI and receptors on the surface of invading tumour cells as follows: Plasmin(ogen) attaches to plasmin(ogen) receptor on the cell surface and it allows the tumour cells to invade extracellular matrix. UTI, exogenously added to the cells, attached to its receptor on the tumour cell surface efficiently inhibits plasmin activity in the close environment of the cells and thus it contributes to prevent tumour cell invasion and metastasis. UTI may play a regulatory role in uPA/plasmin-dependent tumour cell invasion and metastasis. Thus, UTI might provide a therapeutically promising basis for the prevention of tumour metastasis as an anti-metastatic agent.

In general, the clinical application of an agent such as UTI may be fraught with difficulty. It is likely that prolonged administration would be necessary with all the ensuing consequences. The experimental schedules used in this study would not reveal these potential problems. However, the molecule is unlikely to be antigenic, since UTI is therapeutically promising for the treatment of acute pancreatitis in Japan.

With respect to the effect of UTI, our *in vivo* observations in animal models may guide future clinical trials. However, further investigations need to be performed to confirm these preliminary *in vivo* results in other tumour models and to evaluate the clinical side-effects from chemotherapy in combination with UTI for the treatment of human cancer.

**Acknowledgement**

We thank Y Henmi (Nippon Kayaku, Tokyo) for insight into data interpretation.

**References**

ALBRECHT GJ, HOCHSTRASSER K AND SALIER J-P. (1983). Elastase inhibition of the inter-a-trypsin inhibitor and derived inhibitors of man and cattle. *Hoppe-Seyler's Z. Physiol. Chem.*, **364**, 1703–1708.

BALDUYCK M, LAROUI S, MIZON C AND MIZON J. (1989). A proteoglycan related to the urinary trypsin inhibitor (UTI) links the two heavy chains of inter-a-trypsin inhibitor. *Biol. Chem. Hoppe-Seyler*, **370**, 329–336.

BURGERS JK, MARSHALL FF AND ISAACS JT. (1989). Enhanced anti-tumor effects of recombinant human tumor necrosis factor plus VP-16 on metastatic renal cell carcinoma in a xenograft model. *J Urol.*, **142**, 160–164

CAJOT JF, SCHELEUNING WD, MEDCALF RL, BAMAT J, TESTUZ J, LIEBERMAN L AND REICH E. (1989). Mouse L cells expressing human prourokinase-type plasminogen activator: Effects on extracellular matrix degradation and invasion. *J. Cell Biol.*, **109**, 915–925.

CROWLEY CW, COHEN RL, LUCAS BK, LIU C, SHUMAN MA AND LEVINSON AD. (1993). Prevention of metastasis by inhibition of the urokinase receptor. *Proc. Natl Acad. Sci. USA*, **90**, 5021–5025.

DANO K, ANDREASEN PA, GRONDAHL-HANSEN J, KRISTENSEN PI, NIELSEN LS AND SKRIVER L. (1985). Plasminogen activators, tissue degradation, and cancer. *Adv. Cancer Res.*, **44**, 139–266.

GEBHARD W, HOCHSTRASSER K, FRITZ H, ENGHILD J, PIZZO SV AND SALVESEN G. (1990). Structure of inter-α-inhibitor (inter-α-trypsin inhibitor): current state and proposition of a new terminology. *Biol. Chem. Hoppe-Seyler*, **371** (suppl), 13–22.

HEARING VJ, LAW LW, CORTI A, APPELLA E AND BLASI F. (1988). Modulation of metastatic potential by cell surface urokinase of murine melanoma cells. *Cancer Res.*, **48**, 1270–1278.

KOBAYASHI H, OHI H, SHINOHARA H, SUGIMURA M, FUJII T, TERAO T, SCHMITT M, GORETZKI L, CHUCHOLOWSKI N, JANICKE F AND GRAEFF H. (1993). Saturation of tumor cell surface receptors for urokinase-type plasminogen activator by amino-terminal fragment and subsequent effect on reconstituted basement membranes invasion. *Br. J. Cancer*, **67**, 537–544.

KOBAYASHI H, SHINOHARA H, OHI H, SUGIMURA M AND TERAO T. (1994*a*). Urinary trypsin inhibitor (UTI) and fragments derived from UTI by limited proteolysis efficiently inhibit tumor cell invasion. *Clin. Exp. Metastasis*, **12**, 117–128.

KOBAYASHI H, GOTOH J, SHINOHARA H, MONIWA N AND TERAO T. (1994*b*). Inhibition of the metastasis of Lewis lung carcinoma by antibody against urokinase-type plasminogen activator in the experimental and spontaneous metastasis model. *Thromb. Haemost.*, **71**, 474–480.

KOBAYASHI H, GOTOH J, FUJIE M, SHINOHARA H, MONIWA N AND TERAO T. (1994*c*). Effects of urinary trypsin inhibitor on the invasion of reconstituted basement membranes by ovarian cancer cells. *Int. J. Cancer*, **57**, 727–733.

KOBAYASHI H, SHINOHARA H, TAKEUCHI K, ITOH M, FUJIE M, SAITOH M AND TERAO T. (1994*d*). Inhibition of the soluble and the tumor cell receptor-bound plasmin by UTI and subsequent effects on tumor cell invasion and metastasis. *Cancer Res.*, **54**, 844–849.

KOBAYASHI H, GOTOH J, FUJIE M AND TERAO T. (1994*e*). Characterization of the cellular binding site for the urinary trypsin inhibitor. *J. Biol. Chem.*, **269**, 20642–20647.

KOBAYASHI H, FUJII M, SHINOHARA H, ITOH M, TAKEUCHI K, SUGIMURA M, OHI H AND TERAO T. (1995). Inhibition of the metastasis of Lewis lung carcinoma by urinary trypsin inhibitor in the experimental and spontaneous metastasis model. *Jpn. J. Cancer Chemother.* (in press).

LIOTTA LA, RAO CN AND BARSKY SH. (1983). Tumor invasion and the extracellular matrix. *Lab. Invest.*, **49**, 636–649.

MIGNATTI P, ROBBINS E AND RIFKIN DB. (1986). Tumor invasion through the human amniotic membrane: requirement for a proteinase cascade. *Cell*, **47**, 489–498.

OSSOWSKI L AND REICH E. (1980). Experimental model for quantitive study of metastasis. *Cancer Res.*, **40**, 2300–2309.

OSSOWSKI L AND REICH E. (1983). Antibodies to plasminogen activator inhibit human tumor metastasis. *Cell*, **35**, 611–619.

OSSOWSKI L. (1988). In vivo invasion of modified chorioallantoic membrane by tumor cells: the role of cell surface-bound urokinase. *J. Cell Biol.*, **107**, 2437–2445.

OSTROWSKI LE, AHSAN A, SUTHAR BP, PAGAST P, BAIN DL, WONG C, PATEL A AND SCHLTZ RM. (1986). Selective inhibition of proteolytic enzymes in an in vivo mouse model for experimental metastasis. *Cancer Res.*, **46**, 4121–4128.

REICH R, THOMPSON EW, IWAMOTO Y, MARTIN GR, DEASON JR, FULLER GC AND MISKIN R. (1988). Effects of inhibitors of plasminogen activator, serine proteases, and collagenase IV on the invasion of basement membranes by metastatic cells. *Cancer Res.*, **48**, 3307–3312.

SAIKI I, YONEDA J, KOBAYASHI H, IGARASHI Y, KOMAZAWA H, ISHIZAKI, Y, KATO I AND AZUMA I. (1993*a*). Antimetastatic effect by anti-adhesion therapy with cell-adhesive peptide of fibronectin in combination with anticancer drugs. *Jpn. J. Cancer Res.*, **84**, 326–335.

SAIKI I, YONEDA J, IGARASHI Y, AOKI M, KUSUNOSE N, ONO K AND AZUMA I. (1993*b*). Antimetastatic activity of polymeric RGDT peptide conjugated with poly(ethylen glycol). *Jpn. J. Cancer Res.*, **84**, 558–565.

WACHTER E AND HOCHSTRASSER K. (1981). Kunitz-type proteinase inhibitors derived by limited proteolysis of the inter-α-trypsin inhibitor, IV. *Hoppe-Seyler's Z. Physiol. Chem.*, **362**, 1351–1355.